# Bitten to the Bone: A Case of Anxiety-Induced Osteomyelitis

**DOI:** 10.7759/cureus.31511

**Published:** 2022-11-14

**Authors:** Jose A Rivera, Daniel Aragon, Percy M Thomas, Michael Menowsky, Olabiyi O Akala, Paul Dominici, Daniela Hernandez

**Affiliations:** 1 Internal Medicine, Doctors Hospital at Renaissance/University of Texas Rio Grande Valley, Edinburg, USA; 2 Emergency Medicine/Critical Care, Doctors Hospital at Renaissance/University of Texas Rio Grande Valley, Edinburg, USA; 3 Emergency Medicine, Doctors Hospital at Renaissance/University of Texas Rio Grande Valley, Edinburg, USA

**Keywords:** onychophagia, hispanic population, rio grande valley, severe anxiety, osteomyelitis diagnosis

## Abstract

Onychophagia is a habitual nail-biting disorder, usually associated with mental or emotional diseases. It affects 20-30% of the population in all age groups. Human bites have the potential to become serious injuries due to high virulence in the human oral flora and may often require hospital admission, antibiotics and even debridement in the operating room. Thus, repetitive nail biting has the potential to be limb-threatening if not treated early and appropriately.

We present a 49-year-old Spanish-speaking gentleman, with a past medical history of repetitive nail biting secondary to severe anxiety, major depression disorder, bilateral hand neuropathy secondary to diabetes mellitus (DM) type 2 who was initially admitted to the hospital due to cellulitis of the fingers with suspected osteomyelitis in the right hand. Anxiety was being treated by psychiatrist with paroxetine however, given no improvement and prolonged follow-ups, the primary care physician (PCP) added hydroxyzine and scheduled alprazolam in an attempt to minimize symptoms. Despite these efforts, patient continued with nail biting. On initial physical exam, the patient had a lack of fingernails and multiple wounds at various stages of healing across all digits. The distal and middle phalanges of the third right digit showed increased erythema and swelling and band tightening. Patient was started on broad-spectrum antibiotics. Initial radiography of the right hand was concerning for osteomyelitis which was later confirmed with Magnetic Resonance Imaging (MRI). Infectious disease specialist agreed on a course of cefepime, vancomycin and metronidazole. On admission, hand surgeon did not see a need for amputation and patient was treated conservatively. Due to minimal improvement after six days on IV antibiotics, patient underwent fasciotomy of the flexor compartment of the right middle finger after patient rejected hand surgeon's recommendation for amputation. He was discharged to a skilled nursing facility where he was to continue intravenous antibiotics for an additional four weeks.

The vulnerable patient population of South Texas is predominately Hispanic, Spanish-speaking and uninsured. It is imperative to treat psychiatric disorders early to prevent complications, however, given the low numbers of psychiatrists in the Rio Grande Valley and even fewer who speak Spanish it is not unusual to find an appointment in more than six months. In this case, we observe how a trivial everyday behavior can lead to limb-threatening complications if not treated early and appropriately.

## Introduction

Onychophagia is a habitual nail-biting disorder, usually associated with mental or emotional diseases [1-3]. It affects 20-30% of the population in all age groups [3]. Human bites have the potential to become serious injuries due to the high virulence of the human oral flora and may often require hospital admission, antibiotics and even debridement in the operating room [1,3-5]. Thus, repetitive nail biting has the potential to be limb-threatening if not treated early and appropriately [5].

The effective management of onychophagia requires a multidisciplinary approach that involves social support, psychiatric therapy as well as dermatologic treatment and yearly dental visits [3]. The best initial approach involves behavior-modifying techniques such as habit reversal training (HRT) or aversion therapy [1,3]. Patients with underlying psychiatric disorders should have frequent follow-ups with psychiatry in order to explore these behavior-modifying techniques as well as explore pharmacotherapy [3]. Though pharmacotherapy is a second-line treatment for nail biting, drugs such as N-acetylcysteine (NAC), selective serotonin reuptake inhibitors such as fluoxetine and tricyclic antidepressants such as clomipramine have been shown to provide relief [1,3].

From 2003-2013 there was decline of approximately 10% of psychiatrists in the US, which only worsened the already stressed access to psychiatric services in the country [4]. Due to the worsening psychiatric shortage, there are significant delays in treatment, a perceived reduction in the quality of treatment and unacceptable patient experiences [4]. Ultimately this results in poor patient outcomes and higher costs of care.

Here, we present the case of a Spanish-speaking gentleman, dissatisfied with his psychiatric care, who presented with limb-threatening osteomyelitis of a finger due to chronic nail biting secondary to severe anxiety after failing pharmacotherapy. 

## Case presentation

Patient is a 49-year-old Spanish-speaking gentleman with a past medical history of severe anxiety, bilateral hand neuropathy secondary to uncontrolled diabetes mellitus (DM) type 2 and bilateral hand excoriations secondary to repetitive nail biting who was initially referred to the hospital due to worsening cellulitis of the right third finger. Despite treatment with amoxicillin-clavulanate for 10 days, there was no improvement of the cellulitis and x-ray done at the primary care's office was concerning for osteomyelitis.

Patient reported he had chronic nail biting due to his severe anxiety. He had visited several psychiatrists in the past, but felt he was misunderstood due to language barriers and had failed treatment with escitalopram. Psychiatry had switched patient to paroxetine approximately seven months prior to presentation and he continued with an inability to fulfill everyday activities due to anxiety; there was no change in his nail biting. Due to long wait time in order to see a Spanish-speaking psychiatrist, his primary care physician (PCP) placed patient on hydroxyzine and scheduled alprazolam in an attempt to minimize his symptoms. Despite these efforts, patient continued biting his nails and due to his neuropathy from uncontrolled DM type 2 he had little to no pain.

**Figure 1:**
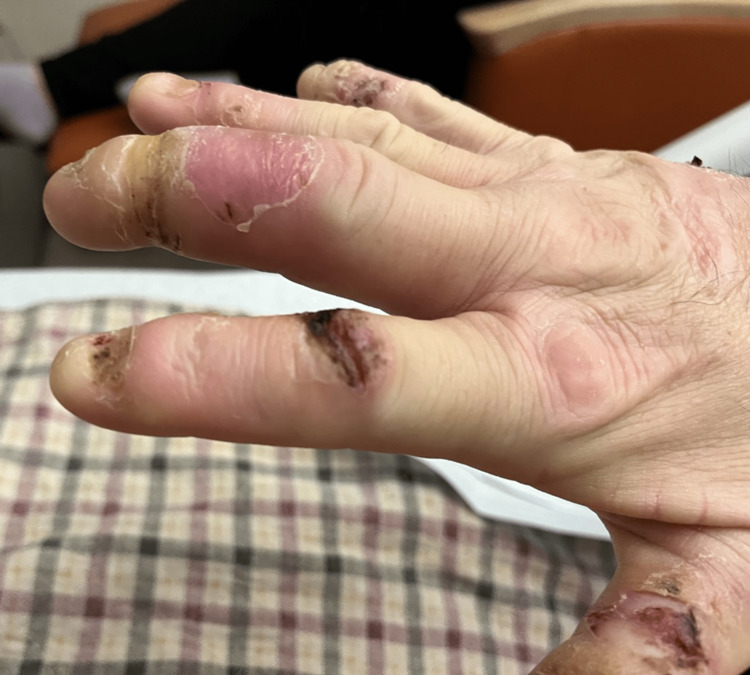
Right hand 24 hours after starting broad spectrum antibiotics. Already with marked improvement from admission.

**Figure 2:**
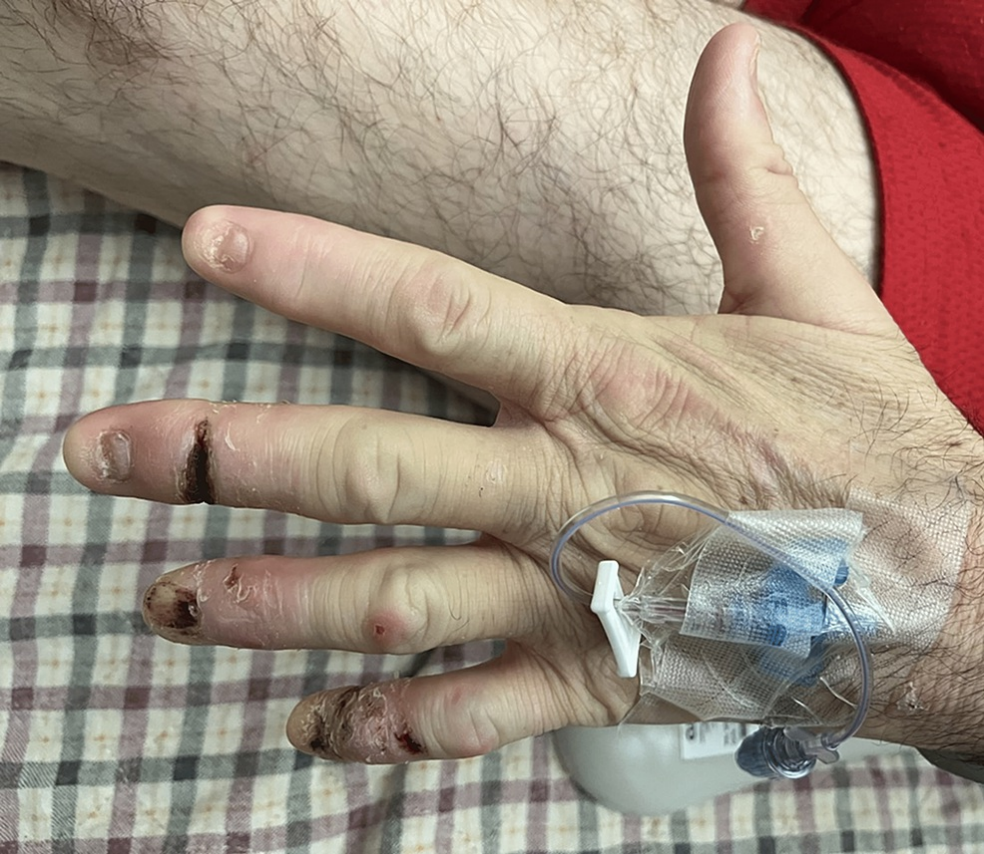
Left hand 24 hours after broad spectrum antibiotics were started. Already with marked improvement from admission.

On the initial physical exam, the patient had a lack of fingernails and multiple wounds at various stages of healing across all digits (Figure 1, 2). The distal and middle phalanges of the third right digit showed increased erythema and swelling with band tightening (Figure 1). Initial labs were unremarkable (Tables 1-3). 

**Table 1:** CBC on admission CBC: complete blood count, WBC: white blood cells, Hgb: hemoglobin, Hct: hematocrit, Plt: platelets

CBC	
WBC	5.3 (4.8-10.9 th/uL)
Hgb	12.2 (10.8-14.7 g/dL)
Hct	37.30% (32.2%- 42.9%)
Plt	292 (146-388 th/uL)

**Table 2:** BMP on admission BMP: basic metabolic panel, Cr: creatinine, BUN: blood urea nitrogen, Glu: glucose

BMP	
Na	134 (132-143 mmol/L)
K	4.0 (3.5-5.1 mmol/L)
Chl	98 (98-107 mmol/L)
CO2	21 (21-31 mmol/L)
Cr	0.9 (0.6-1.2 mg/dL)
BUN	15 (7-25 mg/dl)
Glu	296 (70-113 mg/dl)

**Table 3:** Final blood cultures

Final Blood Culture Results	
Aerobic	Negative
Anaerobic	Negative

**Figure 3:**
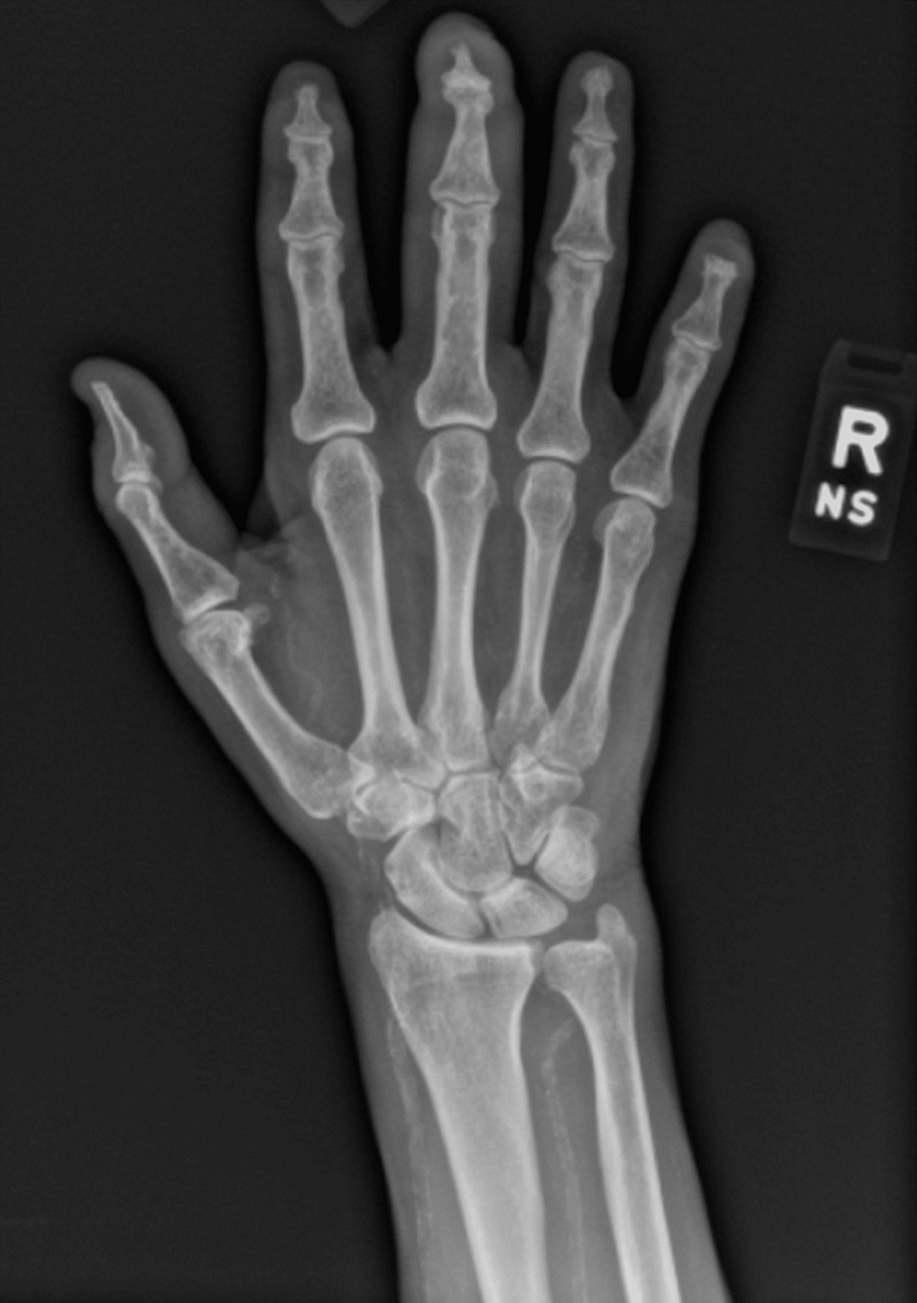
X-ray of right hand. There is absence of the tip of the third digit distal phalanx with a questionable osteomyelitis.

**Figure 4:**
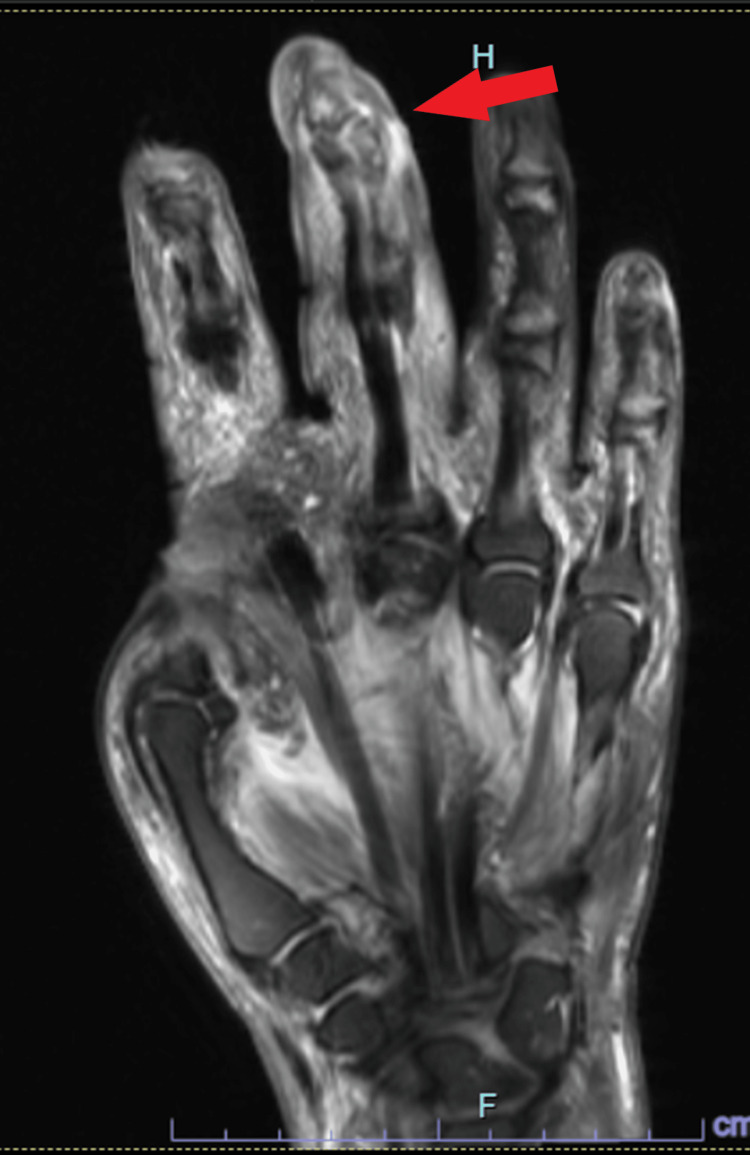
MRI of right hand. Osteomyelitis suspected at about the middle and distal phalanges of the third finger with surrounding cellulitis, myositis with flexor and extensor tenosynovitis.

Patient was started on broad-spectrum antibiotics with vancomycin and pipercillin-tazobactam on admission. Infectious disease specialist agreed on a course of cefepime, vancomycin and metronidazole the day after admission. Initial radiography of the right hand was concerning for osteomyelitis (Figure 3) which was later confirmed with Magnetic Resonance Imaging (MRI) (Figure 4). 

On admission, hand surgeon did not see a need for amputation, unfortunately the patient did require a fasciotomy of the flexor compartment of the right middle finger on day six of admission due to slow recovery. Hand surgery however was not convinced patient would make a meaningful recovery and advocated for amputation of finger; patient decided to continue non-operative treatment. 

After fasciotomy, the patient continued to have marked improvement and was ultimately discharged to a skilled nursing facility with a peripheral inserted central catheter (PICC) where he was to continue intravenous antibiotics for four more weeks. In regard to his psychiatric care, the patient had a psychiatry appointment a few days after discharge and reported concerns of close follow-up due to prolonged appointments. 

## Discussion

The Diagnostic and Statistical Manual of Mental Disorders, Fifth Edition (DSM-5), defines nail biting as a body-focused repetitive behavior and categorized it as "other specified obsessive-compulsive disorders" (OCD). Though the patient presented here did not have a previous diagnosis of OCD, only a diagnosis of major depression disorder and generalized anxiety, the link between anxiety and nail biting is controversial [6]. The American Psychiatric Association define the diagnostic criteria for nail biting as patients who have failed attempts at suppressing the compulsive behavior even in the face of negative social impact [3].

Onychophagia begins in childhood and is usually resolved over time. The prevalence of nail biting between the ages of 17-35 is 21.5% with a bigger decrease in the prevalence after the age of 40 [5]. Though, these numbers are likely underestimated due to reluctance in seeking medical care and can range from 3% - 46.9% among different population groups [7]. Etiology of onychophagia is currently unknown, but genetic, environmental and psychosocial stressors are associated with its onset and severity [1,3,8]. 

The repetitive hand-to-mouth behavior serves as a fecal-oral bridge that may lead to illnesses such as cellulitis, pinworm infections, complications during dental procedures and even osteomyelitis [9,10]. The saliva of chronic nail biters has an increased prevalence of Enterobacteriaceae when compared to those who are not nail biters causing them to have a higher contamination risk [10].

Treatment options are non-pharmacologic and pharmacologic. Non-pharmacological include aversive therapies, competitive stimuli, object manipulation, habit reversal and using bitter-tasting lacquer [11]. Pharmacological therapy includes selective serotonin reuptake inhibitors (SSRIs), N-acetylcysteine (NAC), tricyclic antidepressants (TCAs), dopamine agonists and lithium [1,3]. Treatment of complications is based on severity and center-specific antibiotic guidelines. 

Onychophagia may lead to significant psychosocial problems. Since onychophagia is a challenging disorder to treat, a multi-disciplinary approach involving primary care physicians, psychiatrists, dermatologists and dentists may be required. In the Rio Grande Valley (RGV), located in the southernmost tip of Texas, the prevalence of anxiety and depression has increased while the number of physicians struggles to keep up with demand [4,12-14]. In 2021, an estimated 27.6% of Texans reported an unmet need for counseling or therapy when it came to depression or anxiety symptoms, which was higher than the national average of 26.9% [15]. An estimated 10% of psychiatrists are expected to retire within the next couple of years and the consequences of this will have a pronounced impact in the RGV [4,12-13]. Furthermore, the lack of Spanish-speaking psychiatrists in the country, an estimated 5.5% of all psychiatrist in the United States, create language barriers that can limit access to quality care [15]. Even with the use of an interpreter, Spanish-speaking patients feel less comfortable sharing personal experiences with an English-speaking therapist [15].

## Conclusions

The patient described in this case reported that his chronic nail biting was secondary to his generalized anxiety. He had failed therapy with two different SSRIs and had not previously attempted behavior-modifying therapy. In an attempt to improve his symptoms, PCP prescribed hydroxyzine and alprazolam with no avail. The patient was frustrated due to lack of ability to find Spanish-speaking psychiatrists and the long wait times in between follow-ups. His uncontrolled nail biting led to cellulitis of his finger and eventually osteomyelitis. Though his finger was saved from amputation during this admission, it is difficult to predict if he will continue to self-harm and spread further infection to other fingers unless his psychiatric disorders are not treated accordingly.

The vulnerable patient population of South Texas is predominately Hispanic, Spanish-speaking and uninsured. It is imperative to treat psychiatric disorders early to prevent complications, however, given the few numbers of psychiatrists in the Rio Grande Valley and even fewer who speak Spanish it is not unusual find an appointment more than six months out. The patient population of the RGV deserves improved access to mental health, including Spanish-speaking providers. In this case, we observe the limb-threatening complications trivial everyday behavior can cause if not treated early and appropriately. 
